# A study protocol to evaluate the relationship between outdoor air pollution and pregnancy outcomes

**DOI:** 10.1186/1471-2458-10-613

**Published:** 2010-10-15

**Authors:** Manuel C Ribeiro, Maria J Pereira, Amílcar Soares, Cristina Branquinho, Sofia Augusto, Esteve Llop, Susana Fonseca, Joaquim G Nave, António B Tavares, Carlos M Dias, Ana Silva, Ismael Selemane, Joaquin de Toro, Mário J Santos, Fernanda Santos

**Affiliations:** 1 Centro de Recursos Naturais e Ambiente, Instituto Superior Técnico, Universidade Técnica de Lisboa, Lisboa, Portugal; 2 Centro de Biologia Ambiental, Faculdade de Ciências, Universidade de Lisboa, Lisboa, Portugal; 3 Department of Sociology, Instituto Universitário de Lisboa, Lisboa, Portugal; 4 Department of Environmental Health, Instituto Nacional de Saúde Ricardo Jorge, Lisboa, Portugal; 5 Department of Epidemiology, Instituto Nacional de Saúde Ricardo Jorge, Lisboa, Portugal; 6 Centro de Saúde de Sines, Administração Regional de Saúde do Alentejo, Sines, Portugal; 7 Centro de Saúde de Grândola, Administração Regional de Saúde do Alentejo, Grândola, Portugal; 8 Centro de Saúde de Santiago do Cacém, Administração Regional de Saúde do Alentejo, Santiago do Cacém, Portugal

## Abstract

**Background:**

The present study protocol is designed to assess the relationship between outdoor air pollution and low birth weight and preterm births outcomes performing a semi-ecological analysis. Semi-ecological design studies are widely used to assess effects of air pollution in humans. In this type of analysis, health outcomes and covariates are measured in individuals and exposure assignments are usually based on air quality monitor stations. Therefore, estimating individual exposures are one of the major challenges when investigating these relationships with a semi-ecologic design.

**Methods/Design:**

Semi-ecologic study consisting of a retrospective cohort study with ecologic assignment of exposure is applied. Health outcomes and covariates are collected at Primary Health Care Center. Data from pregnant registry, clinical record and specific questionnaire administered orally to the mothers of children born in period 2007-2010 in Portuguese Alentejo Litoral region, are collected by the research team. Outdoor air pollution data are collected with a lichen diversity biomonitoring program, and individual pregnancy exposures are assessed with spatial geostatistical simulation, which provides the basis for uncertainty analysis of individual exposures. Awareness of outdoor air pollution uncertainty will improve validity of individual exposures assignments for further statistical analysis with multivariate regression models.

**Discussion:**

Exposure misclassification is an issue of concern in semi-ecological design. In this study, personal exposures are assigned to each pregnant using geocoded addresses data. A stochastic simulation method is applied to lichen diversity values index measured at biomonitoring survey locations, in order to assess spatial uncertainty of lichen diversity value index at each geocoded address. These methods assume a model for spatial autocorrelation of exposure and provide a distribution of exposures in each study location. We believe that variability of simulated exposure values at geocoded addresses will improve knowledge on variability of exposures, improving therefore validity of individual exposures to input in posterior statistical analysis.

## Background

During last decades, a growing number of studies concerning the relationship between outdoor air quality and its impact on pregnancy outcomes have been published [1], where an increasing incidence of preterm births and low birth weight among this group of population has been reported [2-4]. These outcomes have been associated to air pollutants such as ozone, particulate matter, carbon monoxide or sulfur dioxide [5-14]. Studies concerning air quality assessment in the Alentejo Litoral highlighted evidences of an association between the degradation of air quality and the industrial air pollutants emitted [15-18]. It is now important to assess if there is an association between outdoor air pollution mixtures and pregnancy outcomes, because this association is not yet investigated in the region.

Semi-ecological design studies are widely used to assess effects of air pollution in humans [19]. In this type of analysis, health outcomes and covariates are measured in individuals and exposure assignments are usually based on air quality monitoring stations data. Therefore, estimating individual exposures are one of the major challenges when investigating these relationships with a semi-ecologic design.

To assess human exposure to outdoor air pollution in an ecological study, measurements are frequently collected from a set of known pollutants in air quality monitoring stations placed in sites previously selected for regulatory purposes. Besides the fact that these sampling sites tend to be selected for their expected relatively high concentrations, the obtained data are time series of instantaneous concentrations values measured on a continuous basis for few pollutants and are sparse in space. In this way, it is difficult to obtain data regarding the exposure to mixtures of pollutants (known and unknown) with high sampling density. In the last years, these constraints have been overcome using lichen diversity biomonitoring programs [20,21]. Lichens (symbiotic organisms consisting of fungi and algae or cyanobacteria) are available almost everywhere on the planet, and have been used to monitor air pollution by several pollutants, particularly sulphur, nitrogen, fluoride, metals, radionuclides, dioxins, PAHs, and also particulate matter [22-26]. As they are long-lived organisms, lichens accumulate pollutants over time, reflecting a long-term exposure (from months up to several years); moreover, lichen diversity tend to decline in polluted areas, as a consequence of the harmful effects of the persistence of pollutants on the lichen physiology. Lichen diversity provides an overall measurement of the air quality, since lichens are exposed to the same complex mixture of pollutants that humans have been exposed to in the previous years. This is of critical importance for health studies, since one of the most difficult tasks is to relate the low pollution levels with medium or long-term effects on health [27]. This was shown by the work of Cislaghi and Nimis [20], where lichen diversity value, used as indicator of air pollution, showed a good correlation with lung cancer mortality in north-east Italy; these authors found that the lung cancer mortality was higher in the areas where the lichen diversity was lower. Furthermore, lichen diversity biomonitoring programs allow the adoption of cost-effective sampling strategies with relatively high density of sampling locations, thus generating more spatially detailed data in order to obtain high resolution maps for outdoor pollution [21]. These spatially detailed data are important to assess the different levels of exposure to pollution between individuals living and/or working at different areas inside the same region.

To assess uncertainty of individual exposures to outdoor air pollution, not much work have been yet published [28]. Standard errors of estimated exposure are one way of assessing exposure uncertainty, however it is difficult if not impossible to derive by analytical methods the effect of this uncertainty on the estimated risk of an health outcome and associated confidence intervals, since the amount of uncertainty varies from location to location [29]. Spiegelman [30] recommends exposure validation methods to assess uncertainty. These methods adjust exposure measurements errors collecting simultaneously data on the exposure surrogate and on a gold standard method of exposure assessment collected within a subsample of the main study population. Baxter and co-authors [31] also use a validation study to reduce traffic-related air pollution exposure misclassification (using indoor concentration measurements in a subset of the study population as gold standard method) to estimate the degree of misclassification and correct for it. A different approach followed in recent years is based on geostatistical simulation. Waller and Gotway [29] used this methodology to a case-control study on association between Very Low Birth Weight (birth weight <1500 gr) and pollution from industrial emissions, and found no significant differences between exposed and unexposed groups. This approach assumes a model for spatial autocorrelation of exposure data and uses Monte Carlo simulations to provide a distribution of exposures in each site and to determine personal variability of the exposure. The set of simulations produced have the same statistical and spatial properties as the original exposure data, which means they reproduce the spatial covariance and histogram, and they honor the observed exposure values. The use of simulations as input to further statistical analysis with multivariate regression models provides therefore a mechanism for quantifying the sensitivity of complex systems to spatial variability [32].

In our study we assess spatial uncertainty of individual exposures using this last approach. This method provides multiple realizations (simulations) of observed data, with reproduction of the observed histogram and spatial covariance while matching for conditioning data (observed values). Each simulation yields a unique value for each location and represents a measure of personal exposure, and the distribution of all values in each location, provides a measure of uncertainty at each location. In the end of the simulation process, we use all simulations as input for statistical analysis with multivariate logistic regression. We assess exposure parameter uncertainty using the empirical distribution of the exposure odds ratio parameters, where its mean and empirical confidence intervals are used as point and interval estimates of true odds ratio. The mean odds ratio represents the factor by which being exposed to air pollution compared to not being exposed, changes the odds of the outcome of interest (low birth weight, preterm birth).

## Methods/Design

### Aim and Study Design

In this study we apply a semi-ecologic study consisting of a retrospective cohort study with ecologic assignment of exposure, to investigate the potential association between outdoor pollution and pregnancy outcomes. Health outcomes and covariates are collected at Primary Health Care Center's (PHCC's) by the research team, from pregnant registries, clinical records data and a specific questionnaire administered orally to mothers involved in this study. We collect exposure data using a lichen diversity biomonitoring program and we do data processing taking into account both lichen species richness and abundance, to achieve a lichen diversity index value (LDV) for each sample site. A spatial geostatistical modeling framework based on a sequential simulation algorithm is applied to the set of LDV values, to derive equally probable maps of lichen diversity values to be used as a surrogate for exposure to outdoor air pollution. Simulated exposures are assigned to pregnant geocoded addresses. Spatial variability of simulated realizations will improve knowledge on variability of assigned exposures, improving therefore validity of personal exposures for posterior statistical analysis with multivariate regression methods. For each simulation, we estimate parameters of logistic regression, using maximum likelihood estimator. In the end, we build an empirical distribution for odd ratio of exposure parameters, to assess a point and confidence interval estimate for odd ratio exposures.

#### Hypothesis of the study

The main purpose of this study is to investigate the hypothesis of an association between the levels of outdoor air pollution and the frequency of low birth weight and preterm births outcomes among Alentejo Litoral residents, in period 2007-2010. To explore this hypothesis we use a lichen diversity biomonitoring program to assess human exposure to outdoor air pollution and we apply a semi-ecological design with uncertainty assessment of exposure using a geostatistical framework.

#### Settings

**Figure 1:**
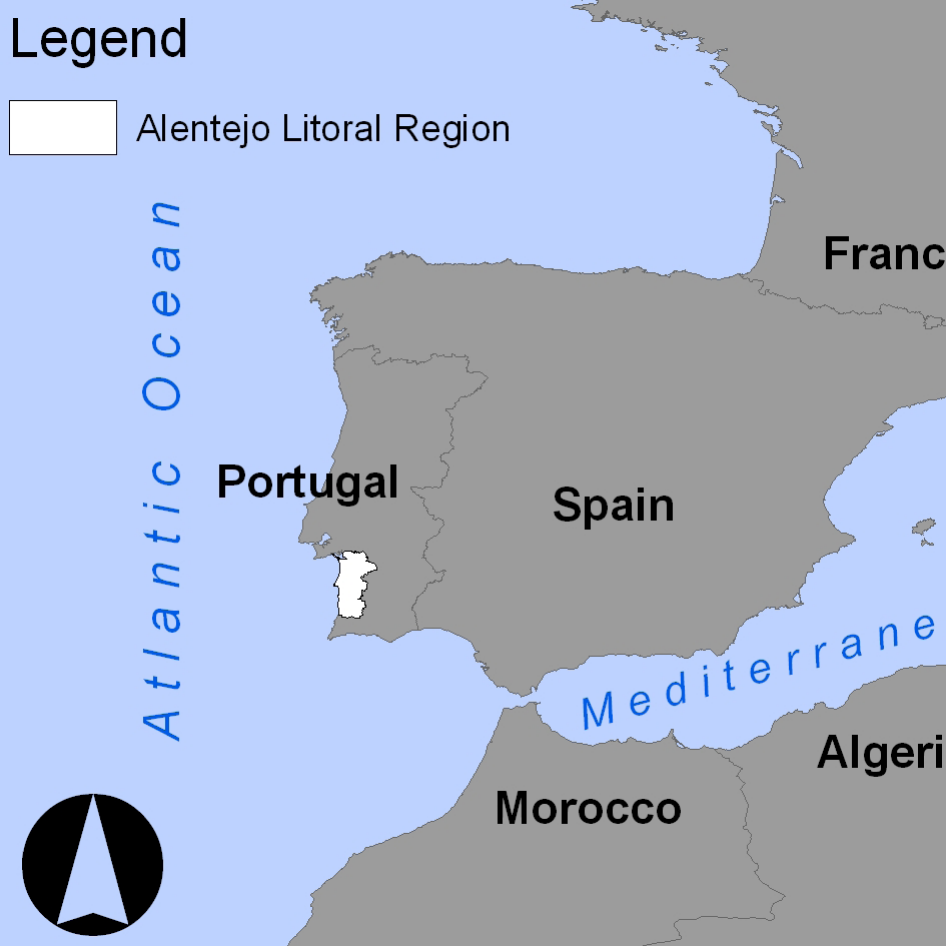
**Alentejo Litoral Region**. Alentejo Litoral Region (white poligon)

The Portuguese Alentejo Litoral region (Figure 1) gathers the administrative areas (Concelhos) of Alcácer do Sal, Grândola, Odemira, Santiago do Cacém and Sines, within a total area of 5300 square kilometers and a resident population of 100 000 inhabitants.

It is mainly located in a coastal region, with several natural parks and other protected areas, and an important industrial area in Sines; this industrial pole comprises mainly petrochemical and energy related industries. A part of study region that includes Santiago do Cacém, Sines and Grândola regions has documented spatial variability of air pollution [17,16,33]. Air pollution seems to be generally higher near urban and industrial areas and there is a trend for better air quality with increasing distance from the sea. There is also an effect of the wind in spreading pollution originating from Sines industrial region to the South, with more disturbed areas occurring in South direction.

The study population is the group of eligible mothers living in this region that accepted to participate in this study. Mothers are eligible if they meet the following inclusion criteria: 1) Pregnant of baby born between 2007 and 2010 in Alentejo Litoral region; 2) Resident in Alentejo Litoral region during pregnancy; 3) Followed by Alentejo Litoral's PHCC's during pregnancy; 4) Singleton births; 5) Didn't use any technique to aid fertilization.

#### Statistical power

Outcome rates for low birth weight and preterm birth in the region for year 2009 are 7% and 8%, respectively. Estimated outcome rates (both low birth weight and premature birth) in pregnant exposed to lower and to upper quartile of LDV are 9% and 5%. Based on these assumptions, the study has statistical power of 80% (with significance criterion level set at 0.05) to detect 4% difference between outcome rates in pregnant exposed to lower and to upper quartile of LDV.

## Materials

### Exposure data

Outdoor air pollution data is assessed through a lichen diversity biomonitoring program.

**Figure 2:**
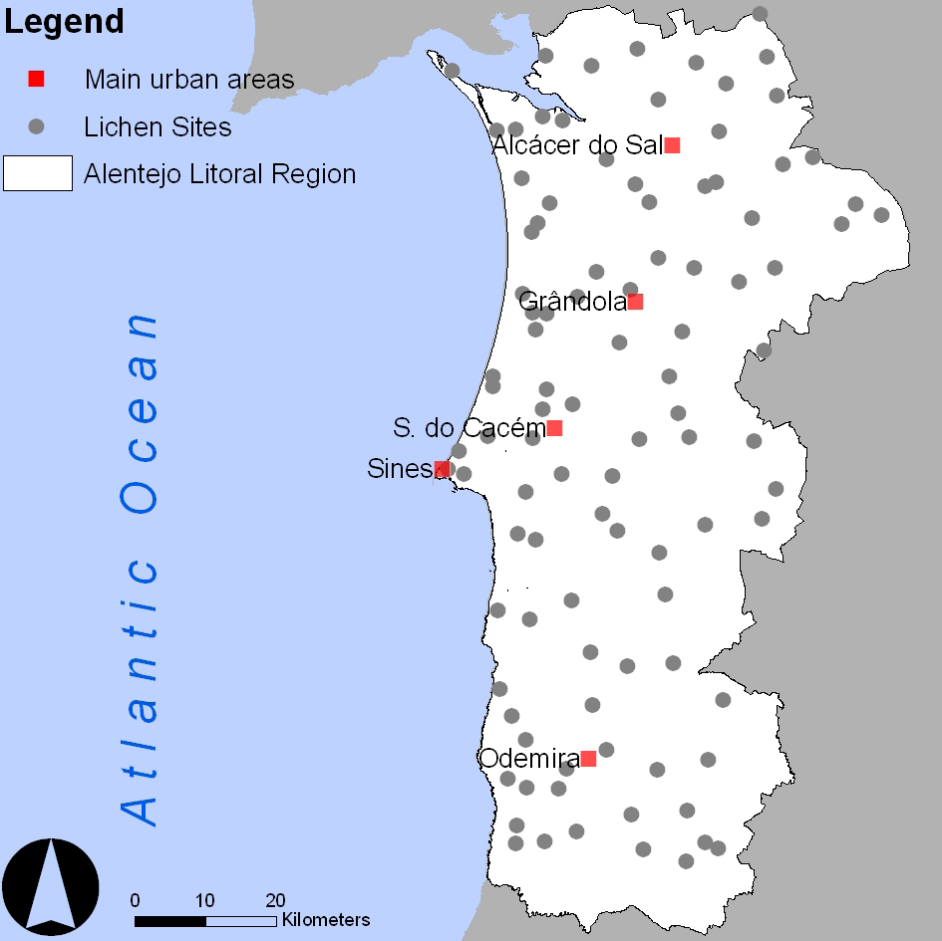
**Sampling sites for outdoor pollution assessment**. Main Urban Areas (red squares), Lichen Sites (grey dots), Alentejo Litoral Region (white polygon)

The number of lichen species and its frequency measured in period 2008-2010 at 98 sites (Figure 2) are used to estimate a Lichen Diversity Value (LDV) index in order to identify the more disturbed areas resulting from air pollution, following a standard protocol according to Asta and co-authors [34].

Approximate site locations are selected in a square grid of 4 km. This spatial resolution is based in previous studies [16,17,21,24] developed in the region using different approaches - LDV, accumulation of pollutants in lichens and diffusion tubes - where the sampling grid resolution has shown to be quite effective in capturing the deposition of most pollutants. The choice of the exact location is based on the following criteria: 1) location covered with *Quercus suber*; 2) Five trees per location (per sample); 3) the same type of landscape namely cork-oak woodlands under constant low-intensity land-use; 4) the highest possible site of the sampling point; and 5) avoidance of sites with local disturbances, particularly main roads or other facilities like farms or small factories. All these conditions are intended to minimize local biodiversity alterations due to local disturbance sources. These criteria may result in underestimation of the local-short distance pollution variability. However, we consider that the spatial resolution adopted is adequate to capture main air pollution spatial patterns.

On each *Quercus suber *tree trunk, a 10 by 50 cm grid with five 10 by 10 cm divisions is used and placed between 120 and 200 cm above ground, in the four main orientations (North, South, East and West). Because both harvested and un-harvested trees are sampled, the grid is always placed on virgin cork. For each orientation, species occurring inside the grid are identified and the numbers of squares in which they occur are recorded as their frequency. The following measurements are recorded for each tree: 1) sampling height (cm), 2) perimeter at breast height (cm), 3) inclination of the tree trunk (degrees), and 4) harvesting status. At each sampling site, further information is also recorded: 1) UTM position, 2) site exposure (N-W-E-S) 3) site location (flat, hill or plateau) 4) altitude (m). Identification of lichen species is mainly done in the field; for those which are not possible to identify in situ, a sample is collected for posterior identification in laboratory. All species are coded with the British Lichen Society recording code numbers [35].

### Health data

Health data is collected at the primary health care network of Alentejo Litoral (PHCC's of Alcácer do Sal, Grândola, Sines, Odemira and Santiago do Cacém) and recorded in a database developed for this project. Estimated number of pregnant residents in Alentejo Litoral region during period of observation is presented in Table 1.

**Table 1:** Target population by administrative area

Administrative area	Population*^1^	Estimated number of pregnants*^2^
Alcácer do Sal	14 287	469

Grândola	14 901	513

Odemira	26 106	775

Santiago do Cacém	31 105	990

Sines	13 577	549

Total	99 976	3 295

^*1^Census 2001

^*2^Estimated from crude birthrates (‰) by Administrative area - National Institute of Statistics (INE), Demographic Indicators

In each PHCC, registered children born since 2007 are tagged. Child's mothers are invited to participate in the study, through an invitation letter with information about the study. A week later, a nurse from research team contacts the child's mother by telephone inviting her once again, to participate in the study and to schedule an appointment at the PHCC. Before questionnaire starts, oral and written information about the project is given to the mother. A written informed consent for participation in this project is asked to the mother. When mothers refuse to participate, written refusal declaration is fulfilled by the nurse.

Before questionnaire starts, mother provides her *Boletim de Saúde da Grávida *(BSG) - a pregnant registry, routinely updated by PHCC's staff (each time the pregnant goes to the PHCC), kept by child's mother, where data on pregnancy outcomes and other events occurred during pregnancy or events occurred during previous pregnancies, pregnant risk exposures (which includes occupational physical activity, other occupational exposures, personal exposures to tobacco in each trimester of pregnancy) and disease family history are registered. Additional information concerning maternal diet, home characteristics, night-time and day-time residence of pregnant, social condition and economic status are collected through a questionnaire designed solely for the purposes of this study.

## Methods

Night and day-time addresses of mothers during pregnancy are georeferenced and gathered in a Geographic Information System (GIS), as are outdoor air pollution data. To estimate individual exposures, geographical coordinates on night-time and day-time addresses during pregnancy are linked to simulated Lichen Diversity Values. Exposure during pregnancy is a weighted average for exposure at day-time and night-time addresses, where we assume 15 hours per day spent at night-time addresses and 9 hours per day spent at day-time addresses.

We compute a LDV for each sampling site, to assess spatial deposition patterns of the pollutants at each location. In order to assess an individual exposure based on LDV sample measures, we estimate a variogram model (a mathematical function used to describe spatial dependence of data) to be used within geostatistical stochastic simulation methods through a sequential algorithm according to Soares [36] in order to create simulated maps. Each simulation is obtained by conditional simulation, conditioned by observed LDV index values, and represents an equally probable realization of spatial distribution of LDV. Therefore, the set of simulations allow us to achieve the objectives of LDV spatial uncertainty assessment and to assign LDV personal exposures.

**Figure 3:**
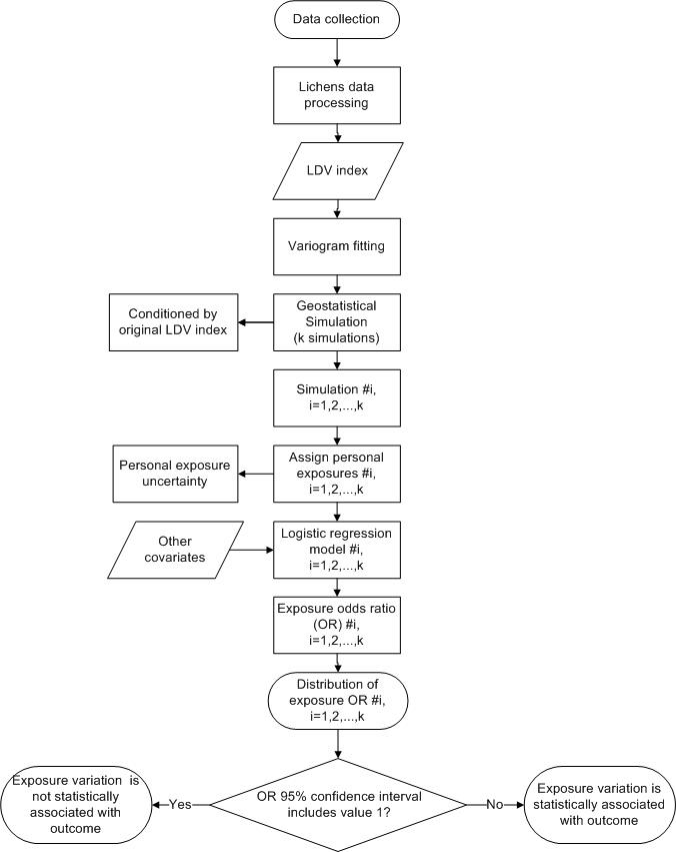
**Diagram describing methodology**.

Both pregnancy outcomes are binomial data, therefore they're modeled via logistic regression. Exposures extracted from each simulated map are used as input data for model fitting, in order to estimate LDV exposure odds ratios while adjusting for other risk factors (see Table 2) such as tobacco smoke (passive or active), residential proximity to air pollution sources (collected as additional information that takes into account local pollution variability), social and economic status, diet, disease family history, drugs or vaccines during pregnancy or complications during pregnancy.

**Table 2:** Other exposure determinants assessment

Other exposure determinants	Assessment
Smoking (active or passive) exposures	Questionnaire

Residential proximity to pollution sources	Questionnaire

Occupational exposures (physical activity and other occupational exposures)	Questionnaire

Social condition and Economic status	Questionnaire

Diet	Questionnaire

Disease family history, drugs, vaccines	BSG

Complications during pregnancy	BSG

For each simulation we fit a logistic regression and we get an odds ratio for LDV exposure. From the set of all simulations we can build an empirical distribution of LDV exposure odds ratio. This enables us to 1) assess a point and a confidence interval estimate for LDV odds ratio and 2) measure how the risk of pregnancy outcome varies with simulated LDV exposures.

### Selection bias

This is a retrospective cohort study where any eligible mother can volunteer to participate in it. Data collection relies on historical data obtained from pregnant registries and questionnaires concerning pregnant risk exposures. Therefore, participation bias is a potential concern.

To reduce possible bias sources, all non-participants are invited to state the reason (or reasons) for not participating. Data on maternal age, parity, place of residence are also collected from clinical files for each non-participant. Differences between non-participant and participant sample population are assessed in what concerns to maternal age, place of residence and parity.

## Discussion

The present study protocol is designed to assess the relationship between air pollution and low birth weight and preterm births outcomes performing a semi-ecological analysis. The originality of the study rest on a global indicator of air quality, the lichen diversity index value (LDV), to allow deriving a continuous metric of exposure, improving the assessment of exposure of each pregnant woman. This study will also use a stochastic method to simulate different (but equally probable) scenarios of each pregnant women exposure to outdoor air pollution, to gain insight into exposure distribution and to assess exposure uncertainty.

One of the main concerns when using lichen diversity to assess exposure of pregnant women is that lichens tend to reflect long-term air pollution (as they are slow-growing organisms) and the impact of air pollution on pregnancy outcomes is assumed to be mainly due to the exposure of women during the pregnancy period itself. However, in pollutants such as bioaccumulative compounds like Dioxins, Furans, Polychlorinated Biphenyls (PCBs), Mercury (Hg), etc. a long-term exposure can also have a negative impact on the pregnancy outcomes, as shown by numerous studies [37-40].

When using lichen diversity to assess the human exposure we are taking benefit of several advantages of using lichens: 1) their index (LDV) reflects the integration of the pollutants over time; however it reflects with more emphasis the recent period of atmospheric deposition; 2) they integrate the biological effect of all pollutants in a synergic way, even of those pollutants that we are not able to measure nowadays, reflecting an integrated environment exposure; 3) The acute and chronic effects of pollutants are also reflected in this biological index; 4) The sampling is flexible in time and space and this allows the construction of more reliable models of air quality, with high spatial resolution to be compared with health data; 5) With those air quality models is possible to establish control areas which is difficult to obtain with any other methodology due to lack of information in space or lack of pollutants analyzed.

The main limitation in the use of lichen index in health studies is that we don't know exactly what specific period of time they are reflecting.

Lichens are very sensitive to sudden pollution episodes (they simply die); the recovery of lichen diversity from an air pollution episode is slowly due to their slow rate of growth. This slow recover does not need to be a problem for time exposure assessment, since in general, after a pollution episode, the pollutants still remain in other components of the ecosystem (soil, water, vegetation, etc.) for a given period of time affecting continuously the human food-chain.

Seasonal patterns of air pollution cannot be assessed using lichen index which might induce misclassification of exposure per trimester of pregnancy. However, in countries of south of Europe, the emission of pollutants remains constant during the seasons, unlike what happens in the north countries where in winter there is an increase of combustion sources. We think that the spatial resolution can be accurately assessed using this method since lichen index is always reflecting the worst pollution conditions independently of the season; the lichens die after the levels of pollutants reach a critical level. Thus, the same criterion is applied in all the studied territory making differences in air pollution index in space comparable.

In summary, this study is designed to assess the relationship between outdoor air pollution and pregnant outcomes performing a semi-ecological analysis. It is a contribution towards the development of new approaches in observational epidemiology because it tackles some limitations of traditional ecological studies, by using LDV index to assess personal exposure and stochastic simulation to assess personal's exposure uncertainty. At this point of the study, outdoor air pollution sample data locations are stored in a geographic information system and lichen biodiversity index are measured for 100% of samples. We found 21% of samples where LDV could not be assessed since no *Quercus suber *trees were available for analysis. We collected data on 628 participants and 70 non-participants. Most part of participants are from Sines (270 participants), 138 are from Grândola, 118 are from Santiago do Cacém, 57 are from Alcácer do Sal and 45 are from Odemira administrative region. The recruitment of participants will continue until April 2011, and if resources are available, until April 2012.

## Ethics committee approval

The study has been approved by the Comissão Nacional de Protecção de Dados (National Committee for Protection of Personal Data). Only fully informed mothers can participate in the study. All participants receive written and oral information about the study, and are asked for their written informed consent.

## Competing interests

The authors declare that they have no competing interests.

## Authors' contributions

MCR is involved in the study design, sample design, health data collection methods, performing geostatistical simulations and statistical analyses described in the protocol and production of this manuscript. MJP conceived the study, participates in study design and coordination, is involved in geostatistical simulations of environmental variables and contributed to the production of this manuscript. AS conceived the study and is involved in geostatistical simulations. CB coordinates all lichens biodiversity analysis and contributed to the production of this manuscript. SA is involved in environmental sampling campaign and lichen biodiversity analysis and had a major effort to the production of this manuscript. EL is involved in environmental sampling campaign and lichen biodiversity analysis. SF is involved in development of questionnaires and interview schemes. JGN is involved in development of questionnaires. ABT and CMD were involved in study design, health data collection, and developing checklist of potential risk factors. AS coordinates health data collection in Primary Health Care Centers of Sines, Alcácer do Sal and Odemira. IS coordinates health data collection in Primary Health Care Centre of Grândola. JT and MJS coordinate health data collection in Primary Health Care Centre of Santiago do Cacém. FS is supervisor for health data collection at Primary Health Care Centers. All authors read and approved the final manuscript.

## Pre-publication history

The pre-publication history for this paper can be accessed here:

http://www.biomedcentral.com/1471-2458/10/613/prepub
